# Quantification of Major Bioactive Constituents, Antioxidant Activity, and Enzyme Inhibitory Effects of Whole Coffee Cherries (*Coffea arabica*) and Their Extracts

**DOI:** 10.3390/molecules26144306

**Published:** 2021-07-16

**Authors:** Boris Nemzer, Diganta Kalita, Nebiyu Abshiru

**Affiliations:** 1 VDF FutureCeuticals, Inc., Momence, IL 60954, USA; Diganta.Kalita@futureceuticals.com (D.K.); Nebiyu.Abshiru@futureceuticals.com (N.A.); 2 University of Illinois at Urbana-Champaign, Urbana, IL 61801, USA

**Keywords:** coffee cherry, extracts, chlorogenic acid, caffeine, antioxidant activity, enzyme, inhibition

## Abstract

Coffee cherry is a rich source of chlorogenic acids (CGAs) and caffeine. In this study we examined the potential antioxidant activity and enzyme inhibitory effects of whole coffee cherries (WCC) and their two extracts on α-amylase, α-glucosidase and acetylcholinesterase (AChE) activities, which are targets for the control of diabetes and Alzheimer’s diseases. Whole coffee cherry extract 40% (WCCE1) is rich in chlorogenic acid compounds, consisting of a minimum of 40% major isomers, namely 3-caffeoylquinic acids, 4-caffeoylquinic acids, 5-caffeoylquinic acids, 3,4-dicaffeoylquinic acid, 3,5-dicaffeoylquinic acid, 4,5-dicaffeoylquinic acid, 4-feruloylquinc acid, and 5-feruloylquinc acid. Whole coffee cherry extract 70% (WCCE2) is rich in caffeine, with a minimum of 70%. WCCE1 inhibited the activities of digestive enzymes α-amylase and α-glucosidase, and WCCE2 inhibited acetylcholinesterase activities with their IC_50_ values of 1.74, 2.42, and 0.09 mg/mL, respectively. Multiple antioxidant assays—including DPPH, ABTS, FRAP, ORAC, HORAC, NORAC, and SORAC—demonstrated that WCCE1 has strong antioxidant activity.

## 1. Introduction

Coffee cherries are one of the world’s most important agricultural commodities and are produced by more than 50 tropical and subtropical countries in the world, with Brazil being the top producer [1]. It is internationally traded as green coffee beans, which are produced by either drying methods under the sun or wet processing [1]. Historically, coffee cherries have long been recognized as great source of nutritional, stimulant, and human health-enhancing properties [2,3,4]. With the differences in culture and tradition, coffee is consumed as brewed coffee, beverages, and supplements with and without roasting. Most coffee beverages consumed worldwide are produced from *Coffea arabica* (Arabica) and *Coffea canephora* (Robusta). *Coffea arabica* is superior due to its sensory properties and, therefore, remains in great demand in the global coffee market.

Oxidative stress is a condition in which an imbalance between the production and neutralization of reactive oxygen species (ROS) exists in the cells and tissues in a biological system. ROS including free radicals such as superoxide anion (O_2_^−^), hydroxyl radical (OH), peroxynitrile (ONOO^−^), and singlet oxygen (^1^O_2_) react with biomolecules such as DNA, lipids, and proteins, causing damage to cellular functions. Cellular dysfunction in turn leads to chronic diseases like cardiovascular diseases, cancer, aging, diabetes, inflammatory diseases, and Alzheimer’s disease [5]. Antioxidants are required to scavenge free radicals and prevent their actions in vivo to protect cells and tissues. Diabetes mellitus is a major metabolic disorder associated with developing insulin resistance, abnormal glucose metabolism, increasing oxidative stress, and developing pathogenic conditions such as neuropathy, retinopathy, and obesity. In humans, two major digestive enzymes, namely pancreatic α-amylase and α-glucosidase, catalyze the hydrolysis of dietary carbohydrates, including starch and oligosaccharides. Inhibition of their activity is an effective way to control postprandial diabetic complications by delaying or suppressing glucose release in the blood stream for absorption in the small intestine [6,7]. Alzheimer’s disease, one of the most common age-related dementias, is a complex irreversible neurodegenerative disorder in which the cholinergic system plays a crucial role. Acetylcholine (Ach) is a low-molecular-weight neurotransmitter. Acetylcholinesterase (AChE) inhibits the action of acetylcholine (Ach) producing acetate and choline. Inhibition of AChE can increase the level and duration of the action of the neurotransmitter [8].

Enzyme inhibitors remain as major target for the development of drugs for treating diabetes and Alzheimer’s disease [8,9,10]. Various drugs such as acarbose and voglibose are clinically approved drugs for the treatment of diabetes. Rivastigmine, tacrine, donepezil, and physostigmine are commercially available drugs for Alzheimer’s disease. However, these commercial synthetic drugs are limited by their short half-lives and severe side effects such as weight gain, hyperglycemia, gastrointestinal disturbances, abdominal distension, flatulence, and hepatotoxicity [11,12]. Therefore, inhibitors from natural plant extracts with negligible or no side effects have gained interest in this regard [7,12].

Coffee cherries are good sources of diverse phytonutrients. Our recent in-depth profiling study of phytochemicals in *C. arabica* identified up to 219 compounds, consisting of isomers of chlorogenic acids (CGAs), flavonoids, alkaloids, eicosanoyl-5-hydroxytryptamide (EHT), atractyligenin, and carboxyatractyligenin derivatives [13]. Caffeine, trigonelline, and CGAs are some of the major compounds present in green coffee beans, with large variations within cultivars. CGAs found in green coffee beans include caffeoylquinic, feruloylquinic, *p*-coumaroylquinic, dicaffeoylquinic, diferuloylquinic, di-*p*-coumaroylquinic, and feruloylcaffeoylquinic acids [14,15,16]. Caffeine is known to increase alertness through stimulation of the central nervous system, blood circulation, and respiration. However, caffeine has some detrimental effects on human health, such as sleeplessness and mild addiction, which has led to the development of decaffeinated coffee products [17]. According to the US Food and Drug Administration, caffeine is generally safe in moderate amounts (<400 mg daily). In addition to their potential as antioxidants, chlorogenic acid-enriched extracts have received considerable attention due to their health benefits, including their association with antidiabetic properties and hepatoprotective, hypoglycemic, and antiviral activities [17,18]. Coffee polyphenol-rich extract significantly improved postprandial hyperglycemia by increasing the release of glucagon-like peptide (GLP-1) and reducing oxidative stress associated with anti-diabetic effects [19]. Consumption of Svetol, a decaffeinated green coffee bean extract with a high CGA content, decreased weight and increased lean/fat ratios in overweight volunteers [20]. A chlorogenic acid-rich extract of coffee cherries, namely “Neurofactor™”, has previously been reported to have a strong antioxidant capacity and increase blood and exosomal levels of brain-derived neurotrophic factor (BDNF), an essential protein for neuronal and brain health [21,22]. Because of the high abundance of chlorogenic acid and caffeine, natural coffee cherry extracts might have a potential role as an alternative to synthetic antidiabetic and anti-Alzheimer’s drug molecules. There are some reports on the antioxidant and antidiabetic properties by coffee brew and extracts. However, the possible inhibition properties of digestive enzyme activities by the whole coffee cherry extracts with specific ratios of CGAs and caffeine are scanty. Therefore, the major objective of our study is to investigate the inhibitory activity of whole coffee cherries and their extracts with specific ratios of CGAs and caffeine on α-glucosidase, α-amylase, and AChE. Another objective of this study is to carry out comprehensive in vitro antioxidant activities adopting multiple antioxidant assays, including DPPH (2,2-diphenyl-1-picrylhydrazyl), ABTS (2,2-azinobis(3-ethyl-benzothiazoline-6-sulfonic acid)), FRAP (ferric reducing antioxidant power), ORAC (oxygen radical absorption capacity), HORAC (hydroxyl radical scavenging capacity), NORAC (Peroxynitrite scavenging capacity), SORAC (superoxide anion scavenging capacity), and SOAC (singlet oxygen scavenging capacity) assays, since a single antioxidant assay may not provide complete information on the antioxidant potential of the coffee extracts.

## 2. Results and Discussion

### 2.1. Identification and Quantification of Major Bioactive Compounds in WCC, WCCE1, and WCCE2

**Figure 1:**
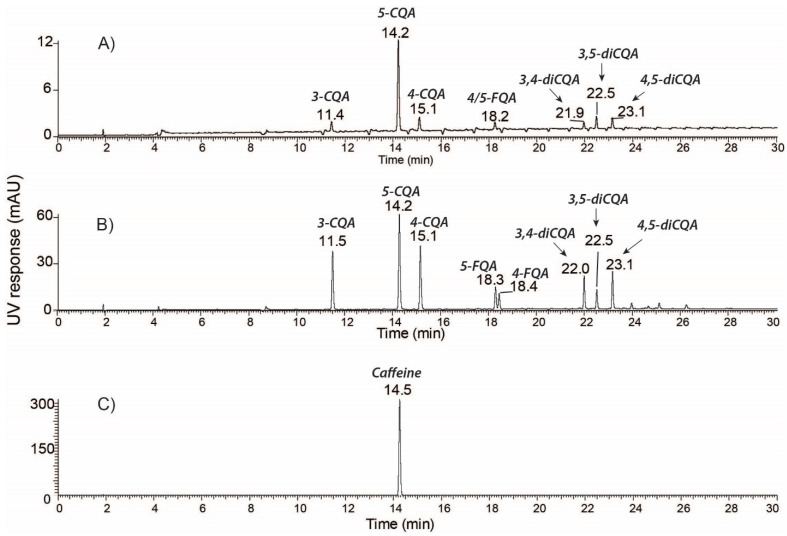
Representative UV-HPLC chromatogram of coffee extracts. CGA profiling in (**A**) WCC powder and (**B**) WCCE1 measured at 325 nm. (**C**) Caffeine profiling in WCCE2 measured at 272 nm. CQA–Caffeoylquinic acid, FQA–Feruloylquinic acid.

**Figure 2:**
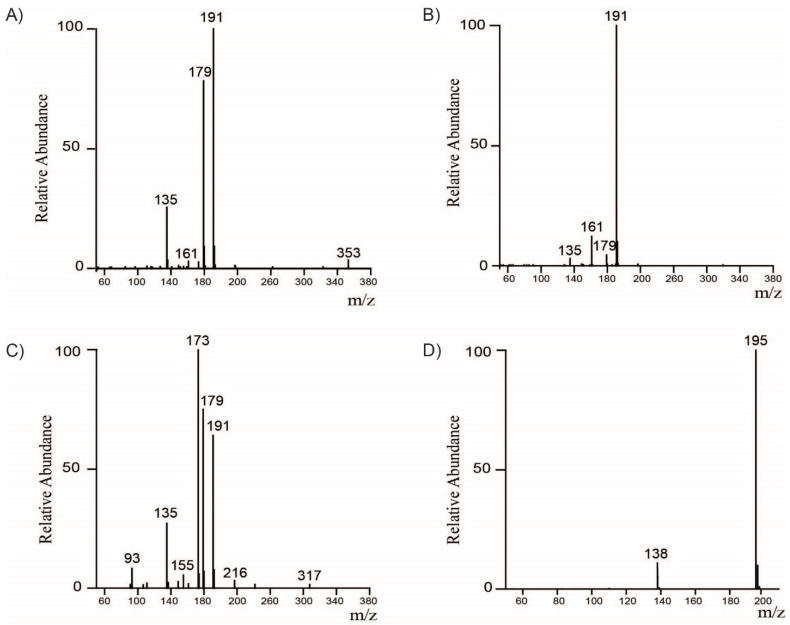
Representative MS/MS spectra of major chlorogenic acids and caffeine. MS/MS spectra of (**A**) 3-CQA, (**B**) 5-CQA, (**C**) 4-CQA, and (**D**) Caffeine. CQA–Caffeoylquinic acid.

Our previous reports and studies from other laboratories have shown that coffee cherries are a rich source of polyphenolic compounds [13,14,15,16]. Various extraction and analytical methods have been used to identify several phenolic compounds, including phenolic acids, flavonoids, alkaloids, and organic acids. In the current study, we quantified the major components of the whole coffee cherry (WCC) and its two commercially available extracts WCCE1 and WCCE2 using HPLC. The UV-HPLC profiles of WCC and WCCE1 are shown in Figure 1A,B. LC-MS/MS analysis of the major chromatographic peaks identified a total of eight chlorogenic acids. Peaks at retention times of 11.4–11.5, 14.2, and 15.1 min were identified as 3-CQA, 5-CQA, and 4-CQA, respectively. Each CQA isoform exhibited unique fragmentation patterns (Figure 2A–C). Dissociation of 3-CQA produced three major fragment ions at *m*/*z* 135, 179, and 191, representing the molecular ions [*caffeic acid*-H-H_2_O]^−^, [*caffeic acid*-H]^−^;, and [*quinic acid*-H]^−^, respectively. Dissociation of 5-CQA generated a major fragment ion at *m*/*z* 191, corresponding to [*quinic acid*-H]^−^;. 4-CQA generated more fragment ions upon dissociation—the major fragments include *m*/*z* 135, 173, 179, and 191, representing the molecular ions [*caffeic acid*-H-H_2_O]^−^, [*quinic acid*-H-H_2_O]^−^, [*caffeic acid*-H]^−^, and [*quinic acid*-H]^−^, respectively. Following a similar approach, we identified two FQA isoforms: 5-FQA (*m*/*z* 367) and 4-FQA (*m*/*z* 367) corresponding to the UV-HPLC peaks at RT 18.2/18.3, and 18.4 min, respectively; and three diCQA isoforms: 3,4-diCQA (*m*/*z* 515), 3,5-diCQA (*m*/*z* 515) and 3,4-diCQA (*m*/*z* 515) corresponding to the UV-HPLC peaks at RT 21.9/22.0, 22.5, and 23.1 min, respectively. Moreover, the major peak at RT 14.5 min in the WCCE2 UV-HPLC chromatogram (Figure 1C) was identified as caffeine by positive ion mode MS/MS analysis. Dissociation of caffeine produced a fragment ion at *m*/*z* 138 (Figure 2D), corresponding to the neutral loss of methyl isocyanate, [caffeine + H–O=C=NCH_3_]^+^. The major MS/MS peak at *m*/*z* 195 corresponded to the undissociated caffeine molecular ion [caffeine + H]^+^.

Quantitative data for the major identified compounds in WCC, WCCE1, and WCCE2 (expressed as % *w*/*w*) are shown in Table 1. The total amounts of CGAs in WCC, WCCE1, and WCCE2 were 6.76 ± 1.34, 46.46 ± 0.93, and 6.1 ± 0.01 % *w*/*w*, respectively. 5-CQA was the major CGA found in all samples. The caffeine level in WCCE2 was 73.60 ± 0.65 % *w*/*w* The caffeine levels were very low in WCC and WCCE1 (Table 1). The amount of trigonelline was found to be much higher in WCCE1 than in WCC and WCCE2 (Table 1). This result suggests that the extraction and purification processes involved in the preparation of WCCE1 indirectly favor trigonelline, whereas the preparation of the caffeine-rich sample, WCCE2, disfavors trigonelline.

### 2.2. Antioxidant Activities

#### 2.2.1. DPPH Radical Scavenging Activity

**Figure 3:**
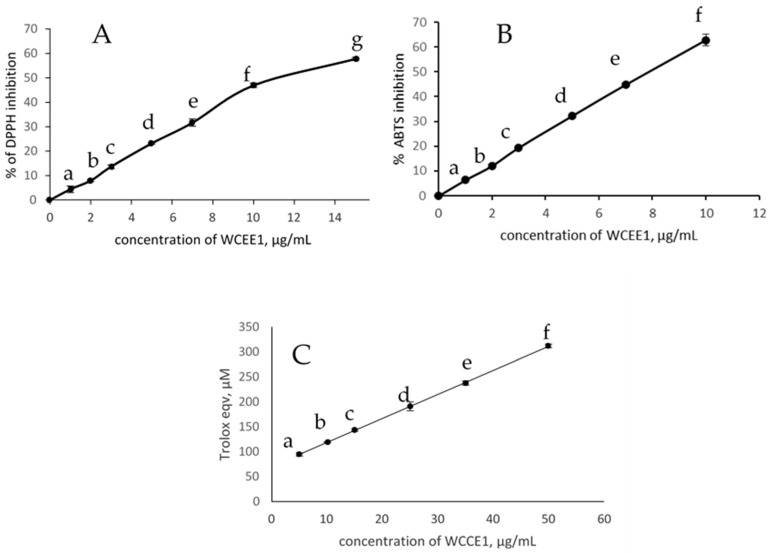
(**A**) DPPH radical scavenging activity, (**B**) ABTS radical scavenging activity, (**C**) Ferric reducing antioxidant power of WCCE1. Values represent mean ± SD of three replicates. Different letters in the data (a, b, c, d, e, f, g) against each concentration indicate that they are significantly different (*p* < 0.05).

The DPPH radical scavenging assay is a very common method used to screen the potential antioxidant activity of synthetic and natural products. It is a stable free radical with an unpaired electron that displays maximum absorption at 515 nm and loses its absorption upon interacting with antioxidants. The advantage of DPPH is that it is an easy, economic, and rapid method to evaluate the radical scavenging activity of non-enzymatic antioxidants to measure the nature of the antioxidants. In our assay, WCCE1 showed the highest antioxidant activity, followed by WCC. WCCE2 did not show DPPH scavenging activity at the concentrations used in the assay. The scavenging activity was observed to occur in a dose-dependent manner (*p* < 0.05) (Figure 3A) with IC_50_ values of 11.2 and 300.0 μg/mL, respectively (Table 2). In the DPPH assay, antioxidants react with free radicals via different mechanisms: hydrogen atom transfer (HAT), a single electron transfer (SET) mechanism, or a combination of both HAT and SET. However, a limitation of this assay with the coffee samples is that the color of the coffee sample might interfere with DPPH absorption. Furthermore, DPPH is not the most physiologically relevant radical.

#### 2.2.2. ABTS Radical Scavenging Activity

The ABTS assay utilizes the free radical, ABTS, generated when the substrate is oxidized with potassium persulfate. ABTS is a blue/green color with a maximum absorbance at 734 nm in water. The radicals are decolorized in the presence of antioxidants. An advantage of the ABTS assay is that it can be used at different pH values. It also takes care of the lipophilic and hydrophilic characteristics of the antioxidants. Similar to the DPPH assay, ABTS scavenging activity occurred in a dose-dependent manner (*p* < 0.05) for WCCE1 (Figure 3B) and WCC, with IC_50_ values of 7.85 and 140.0 µg/mL respectively (Table 2). WCCE2 did not exhibit ABTS scavenging activity within the tested concentration range.

#### 2.2.3. Ferric Reducing Power Activity (FRAP)

The FRAP assay is a nonspecific, redox-linked colorimetric assay that is related to the molar concentration of antioxidants present. It requires a low pH to reduce the ferric-TPTZ to ferrous ions, which show a maximum absorbance at 593 nm. The increase in absorbance at 593 nm is proportional to the ferric reducing power of the test sample. It is a highly reproducible and inexpensive method to screen the antioxidant potential of different synthetic and natural antioxidants. Significant reducing power was observed for WCCE1 and WCC in a dose-dependent manner (Figure 3C), reflecting the trend in the antioxidant capacity of the DPPH and ABTS radicals. A limitation of FRAP is that it considers recycling capacity and does not include potential antioxidants that work via hydrogen atom transfer (HAT).

#### 2.2.4. ORAC 5.0 Assay Antioxidant Capacity

Different assays use radicals of a specific nature and reactivity. ORAC 5.0 assays interact with a range of free radical species such as peroxyl, hydroxyl, peroxynitrite, superoxide anion, singlet oxygen, viz., ORAC, HORAC, NORAC, SORAC, and SOAC. These assays provide comprehensive antioxidant activities using antioxidants covering both the hydrophilic and hydrophobic antioxidants present in coffee samples. The ORAC method is based on the peroxyl radicals generated from AAPH. It reflects the antioxidant capacity area under the fluorescence decay curve to be determined at fixed time and measures the free radical scavenging rate and reaction kinetics. In the HORAC and ORAC assays, WCCE1 showed the highest ORAC activity, attributable to peroxyl, hydroxyl, and singlet oxygen scavenging capacity, with a total ORAC value of 30,365 µmol TE/g (Trolox Equivalent/gram dry weight). Relatively less scavenging activity was observed with NORAC (Table 3). WCC showed up to 2028 µmol TE/g. Moreover, WCCE2 did not show scavenging activity in the ORAC assay, and very little scavenging activity was observed in the HORAC and NORAC assays.

Coffee beans are a complex mixture of compounds—including organic acids, phenolic acids, alkaloids, and flavonoids—that contribute to the antioxidant activities. However, depending on the variety, pre- and post-harvest conditions, and processing methods, the levels of these compounds vary. Previous studies have reported that CGAs, the major constituents of coffee cherries, are one of the strong natural antioxidant compounds [23]. They mainly cover the hydrophilic antioxidant activity contributed from the in vitro antioxidant assay. CGAs are reported to be strong hydroxyl and superoxide scavengers where the hydroxyl group from phenolic acid donates a hydrogen atom to free radicals [24]. The peroxyl radical scavenging ability of 5-CQA was also reported to be strong [23]. Structure–activity relationship studies have demonstrated that the number and spatial differences of functional groups in the antioxidants influence their antioxidant capacity. For example, Xu et al. [25] found that diCQA has a higher antioxidant potential than its mono-caffeoyl units, which is attributed to its higher number of hydroxyl groups. In a number of studies, it was found that scavenging of the activities of hydroxyl radicals is directly proportional to the CGA content. Chlorogenic acid was reported to have strong DPPH scavenging activity, with IC_50_ values of 3.09 and 51.23 µg/mL respectively [26,27]. Mullen et al. [15] reported that chlorogenic acid-rich coffee cherry extract possessed a total ORAC antioxidant capacity of up to 64,354 µmol TE/g.

### 2.3. Inhibition of α-Glucosidase Activity, α-Amylase, and AChE Activities

Alpha glucosidase, located at the brush-border surface membrane of intestinal cells, is the key enzyme that catalyzes the final step in the hydrolysis of carbohydrates and releases glucose from disaccharides and oligosaccharides. Controlling the activities of this enzyme plays a crucial role in the regulation of blood glucose. These activities were monitored by in vitro assays using different combinations of enzyme sources and substrates.

**Figure 4:**
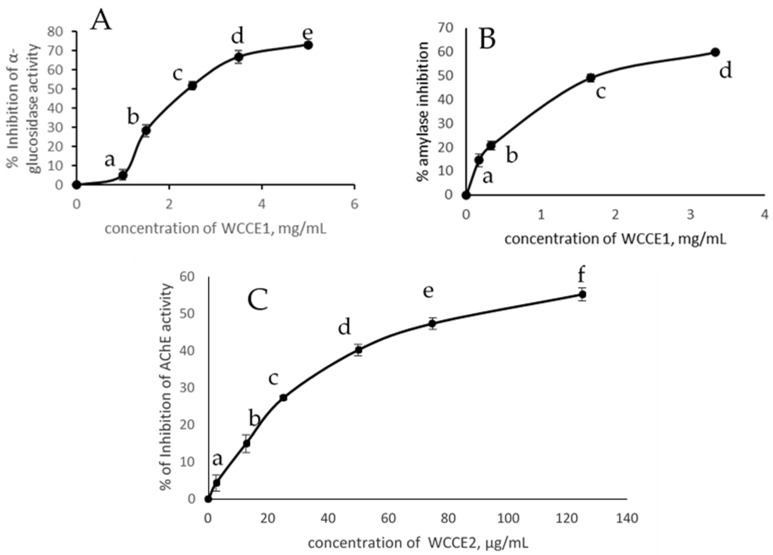
Inhibition of (**A**) α-glucosidase and (**B**) α-amylase activities by WCCE1, and (**C**) Acetylcholinestarase activity by WCCE2. Values represent mean ± SD of three replicates. Different letters in the data (a, b, c, d, e, f) against each concentration indicate significant differences from each other (*p* < 0.05).

In the present study, we tested the potential inhibitory effects of α-glucosidase using pNPG as substrates with and without WCC, WCCE1, and WCCE2. WCCE1 inhibited α-glucosidase activity in a dose-dependent manner (*p* < 0.05) (Figure 4A). WCCE2 did not show any inhibitory against α-glucosidase effects in our assays within the tested concentration range. WCC showed an inhibitory effect at higher concentrations compared to WCC1 (Table 4). The minimal 50% inhibition of glucosidase for WCCE1 was 2.42 mg/mL, which is 12 times lower than that of WCC (Table 4). As mentioned before, WCCE1 is enriched with chlorogenic acid and the major phenolic compound in this extract is 5-CQA; we were interested in determining the inhibitory effect of 5-CQA and found an IC_50_ value of 2.1 mg/mL. The comparable IC_50_ of CGAs and WCCE1 (with 48% of CGAs) indicated that other chlorogenic acids also play an additive role in the inhibition of α-glucosidase.

Chlorogenic acids and their derivatives isolated from coffee and other natural products have been investigated in many studies because of their potential role in the inhibition of α-glucosidase activity. Xu et al. [28] isolated a polyphenol extract from *IIex kudingcha* that contained 74.3% chlorogenic acid derivatives, comprising of 3-CQA, 4-CQA, 5-CQA, 3,4-diCQA, 3,5-diCQA, and 4,5-diCQA, and reported the IC_50_ as 0.42 mg/mL from the inhibitory effects on the activities of α-glucosidase. The purified fractions of the CGA derivatives had a range 0.16–0.39 mg/mL, where 5-CGA had an IC_50_ of 0.30 mg/mL. The chlorogenic acid-rich extract obtained from *Echinacae purpurea* flower extract exhibited a strong inhibitory effect, where the IC_50_ of chlorogenic acid was reported be 0.90 mg/mL for glucosidase [29]. Alongi et al. [30] reported the IC_50_ as 2.41–3.24 mg/mL in coffee brews prepared from green coffee beans, *C. canephora var. robusta*. In another report, they tested α-glucosidase inhibition activity from the brews prepared from roasted and unroasted coffee and found an IC_50_ of 0.50–0.56 mg/mL [31]. The IC_50_ for acarbose was 0.09 mg/mL. CGAs and melanoidins were described as the major inhibitors of this enzyme activity, in support of the antidiabetic potential of coffee consumption [31]. The α-glucosidase inhibitory capacity resulted from the composition of the coffee samples with different degrees of roasting. CGAs represented 77% of phenolic compounds in the sample, followed by di-chlorogenic acids (di-CGA, 22%), which were (both) responsible for glucosidase inhibition activity [31]. In another study, Duangjai et al. [32] reported that 5 mg/mL coffee cherry extract inhibited up to 28.85% of the glucosidase activity. The active compounds—namely ferulic acid, maleic acid, citric acid, caffeic acid, and chlorogenic acid—inhibited glucosidase activity by up to 46.57% at a concentration of 5 mM (1.78 mg/mL). Chlorogenic acid and its synthetic acetyl derivative showed significant inhibition of glucosidase activity at 2.8 mM (1 mg/mL) [33]. Herawati et al. [34] investigated the α-glucosidase inhibitory activity in *C. robusta* coffee beans and reported that the coffee brew extract showed a reduction in glucosidase activity of 69.41% at a concentration of 12.5 g/100 mL concentration [34,35]. The bioactive compounds responsible for antioxidant and anti-α-glucosidase activities were proposed to be phenolic acids.

Alpha amylase (1,4-α-d-glucan-glucanohydrolase, EC 3.2.1.1) is another primary digestive enzyme that catalyzes the hydrolysis of α-1-4-glycosidic linkages of starch and converts starch to its oligosaccharides. Inhibition of α-amylase can be considered a strategy for the treatment of disorders of carbohydrate metabolism by controlling their rate of release. Using human salivary and pancreatic α-amylase and various substrates such as starch and p-nitrophenol derivatives, inhibitors have been screened. We tested the potential anti-amylase activity of coffee cherries and their extracts using human salivary α-amylase. WCCE1 and WCC effectively inhibited amylase activity in a dose-dependent manner (*p* < 0.05). WCCE1 inhibited amylase activity with an IC_50_ of 1.7 mg/mL and WCC had an almost eight times higher IC_50_ (similar α-glucosidase activity) in the extracts. WCCE2 was not an effective inhibitor of amylase activity in our assay.

Few studies have demonstrated that natural CGA-containing extracts strongly inhibit α-amylase activity. Narita and Inouye isolated nine different isomers of CGAs from green coffee beans and investigated their effects on the inhibition of amylase [36]. The IC_50_ values ranged from 0.02 mM to 26.5 mM, where di-caffeoylquinic acid was found to be a stronger inhibitor. The IC_50_ values for 5, 3, 4-CQA were 0.08 mM, 0.23, and 0.12 mM, respectively. In another study, Narita et al. [37] reported that 5-CQA, caffeic acid, and quinic acid inhibited porcine pancreas α-amylase with IC_50_ values of 0.08 mM, 0.40 mM, and 26.5 mM, respectively [36]. The higher inhibitory effect of di-CQA than CQA on α-amylase activity suggests that inhibitory properties increase with an increasing number of caffeic acid (CA) sub-structures [36,37]. Moreover, the inhibitory activities of CA derivatives were always higher than those of ferulic acid derivatives, suggesting that the two neighboring hydroxyl groups on the catechol ring were effective in the inhibition. Zheng et al. [38] examined the inhibitory effect of chlorogenic acid against porcine pancreatic amylase and potato starch as substrates and obtained an IC_50_ of 0.498 mg/mL and acarbose of 2.28 mg/mL. Different results were observed with inhibition assays because of the physicochemical properties of the substrates, which could affect the affinity to the active site of the enzymes [39]. It is now well established that the use of different enzyme sources for inhibition assays and different substrates can yield very different results [40,41]. The chlorogenic acid-rich extract obtained from *Echinaca purpurea* flower extract exhibited a strong inhibitory effect, with an IC_50_ of 1.71 mg/mL for amylase [29]. Coffee brew extract at 5 mg/mL inhibited up to 49.32% of α-amylase activity and its constituents; caffeic acid and chlorogenic acid inhibited 93.35% and 50%, respectively [32]. Nyambe-Silavwe et al. [40] reported that chlorogenic acid and phenolic acids are weak inhibitors of human salivary amylase, as they showed 20% inhibition at 5 mM.

AChE catalyzes the hydrolysis of the neurotransmitter acetylcholine into choline and acetate. The rate of hydrolysis of AChE in *E. electricus* with DTNB produces thiocholine, which was used to screen the potential anticholinesterase activity of WCC, WCCE1, and WCCE2.

In our assay, WCCE2 inhibited AChE activity in a dose-dependent manner in the tested range (0–125 µg/mL) (*p* < 0.05). WCCE1 and WCC did not inhibit AChE activity at the concentrations used in the assay. Figure 4C shows the inhibitory pattern of WCCE2, and the IC_50_ was determined to be 90 µg/mL (Table 3). Physostigmine is a strong commercial inhibitor of AChE that showed nearly complete inhibition at 5 µg/mL (data not shown). We quantified the level of caffeine and found it to be 73%, which demonstrates that the inhibition of AChE occurred due to the presence of caffeine in WCCE2. Synthetic pure caffeine supported this hypothesis, which showed an IC_50_ of 65 µg/mL in our assay. In their study, Pohanka et al. [42] reported caffeine to be a moderate inhibitor of AChE activity, with an IC_50_ of 87 µM. Caffeine inhibits AChE in a noncompetitive manner. The differences in the results from various studies have been found to be due to the sources of AChE and the assay used [43]. Small structural alterations in AChE from different organisms can be responsible for the difference in results [43]. In one study [44], chlorogenic acid showed a moderate inhibitory effect on AChE activity. Although CGAs are a major component of WCCE1, the concentrations used in our assay may not be sufficient to inhibit the activity of AChE.

In the present study, we found a positive correlation between chlorogenic acid content and antioxidant, α-amylase inhibition, and α-glucosidase inhibition, whereas caffeine had a strong positive correlation with AChE inhibition.

## 3. Materials and Methods

### 3.1. Materials and Chemicals

Coffee cherries and its extracts: Whole coffee cherry (*C. arabica*) (WCC) was grown in Chickmaglur, India, and was dried and ground to powder. WCCE1 and WCCE2 were prepared from whole coffee cherries and were commercially marketed as Coffeeberry^®^ by VDF FutureCeuticals, Inc. (Momence, IL, USA). WCCE1, commercially marketed as “Neurofactor™”, was produced using a proprietary multi-step 70% ethanol/water extraction and purification method, followed by spray-drying [13]. WCCE2, commercially marketed as “Coffeeberry^®^ Energy 70%”, was prepared by using a proprietary water extraction and purification procedure and was subsequently spray-dried [13].

Chemicals: DPPH (2,2-diphenyl-1-picrylhydrazyl), ABTS (2,2-azinobis(3-ethyl-benzothiazoline-6-sulfonic acid)), potassium persulfate, FeCl_3_.6H_2_O, TPTZ (2,4,6-tripyridyl-s-triazene), AAPH (2,2′-azobis(2-amidinopropane) dihydrochloride), Fluorescein, Trolox (6-hydroxy-2,5,7,8 tetramethylchroman-2-carboxylic acid), α-glucosidase from *saccharomyces cereviciae*, *p*-Nitrophenyl-α-d-glucoside (pNPG), α-amylase, Acetylcholinesterase inhibitor screening kit, Acetylcholinestarase from *E. electricus*, 5-caffeoylquinic acid, caffeine, Physostigmine, Acarbose, and other chemicals were purchased from Sigma Aldrich Corp. (St. Louis, MO, USA). The α-amylase inhibitor screening kit was purchased from BioVision Inc. (Milpitas, Agilent, CA, USA). Hydroethidine fluorescent stain (5 ethyl-5,6-dihydro-6-phenyl-3,8-phenanathridinediamine) was purchased from Polysciences, Inc. (Warrington, PA, USA).

### 3.2. Identification of Compounds by LC-MS/MS Analysis

LC-MS/MS analysis was performed as described previously by Nemzer et al. [13]. LC-MS/MS analysis was performed on a Q-Exactive Hybrid Quadrupole-Orbitrap mass spectrometer (Thermo Scientific, Waltham, MA, USA) coupled to a Dionex UltiMate 3000 UHPLC system (Thermo Scientific). A C18 analytical column (Agilent Poroshell 120 EC-C18, 3 × 150 mm, 2.7 µm, Agilent Technologies, Santa Clara, CA, USA) was employed for the separation of the compounds as follows: 0–15 min, 7% B; 40 min, 50% B; 50 min, 70% B; 51 min, 0% B and kept for an additional 10 min at 0% B. Flow rate was 0.4 mL/min for 60 min. The MS instrument operated in both positive and negative ion modes with a capillary voltage of 3.2 kV. Precursor ions were scanned in the range of *m*/*z* 150–1200 at a resolution of 70,000 and an automatic gain control target value of 1.0 × 10^6^. Precursor ions were fragmented in the higher-energy collisional activated dissociation cell, and the fragments were analyzed using an orbitrap analyzer. The major chlorogenic acids in each sample were identified manually based on their precursor mass-to-charge ratios and unique fragmentation spectra.

### 3.3. Quantification of Chlorogenic Acids by High-Performance Liquid Chromatography (HPLC)

Chlorogenic acids were quantified using the Agilent 1200 high performance liquid chromatograph (HPLC) system equipped with a gradient pump, a refrigerated autosampler, column heater, PDA detector at 325 nm, and an electronic data system. Samples were prepared by weighing approximately 35 mg (for WCCE1 and WCCE2), and 500 mg (for WCC) into a 50 mL centrifuge tube. Then, 25 mL of 50% aqueous methanol solution was added and sonicated for 10 min using an ultrasonic bath, followed by the addition of 25 mL of the assay diluent, then mixed well and centrifuged. The supernatants were transferred to vials for injection. For standards, approximately 1 mg of chlorogenic acid (5-CQA) standard was added to a 10 mL volumetric flask. The standard solution was diluted to about half volume with the assay diluent, sonicated for 10 min using an ultrasonic bath, followed by the addition of 5 mL of the assay diluent, then mixed well and centrifuged. The analytes were separated by injecting 10 µL into the system and using the Phenomenex Luna (Phenomenex, Torrance, CA, USA) 5 µm C18(2) 100 Å, 150 × 4.60 mm column, and mobile phase A (2% acetic acid) and B (acetonitrile) with the following gradients: 0–9 min, 95% A, 5% B; 9–21.5 min, 91% A, 9% B; 21.50–60.0 min, 75% A, 25% B; 75–85 min, 95% A, 5% B; 85–90 min, 95% A, 5% B, at a flow rate of 1.0 mL/min for 90 min. The concentration of each CGA isomer in the WCC, WCCE1, and WCCE2 samples was calculated as 5-CQA equivalent using the standard concentration and area under the curve (AUC) of the 5-CQA standard. The total CGAs was calculated as the sum of the main CGA isomers: 3-CQA, 5-CQA, 4-CQA, 5-FQA, 4-FQA, 3,4-diCQA, 3,5-diCQA, and 4,5-diCQA.

### 3.4. Quantification of Caffeine and Trigonellin by HPLC

Caffeine was quantified using the HPLC Agilent 1260 system equipped with a gradient pump, a refrigerated autosampler, a column heater, a PDA detector at 275 nm, and an electronic data system. The analytes were separated by injecting 10 µL samples into the system and using the Supelco Escentis Express Phenyl Hexyl column (Supelco Inc., Bellefonte, PA, USA), 150 mm × 3 mm × 2.7 µm, and mobile phase 90:10 of 0.1% perchloric acid at a flow rate of 0.6 mL/min for 15 min. The concentrations of caffeine and trigonelline in the WCC, WCCE1, and WCCE2 samples were calculated based on external standard curves obtained for pure caffeine and trigonelline.

### 3.5. Measurements of Antioxidant Activity

#### 3.5.1. ABTS Radical Scavenging Activity Assay

The ABTS radical scavenging assay was performed following the methods described by Arnao et al. [45]. For the assay, 8 mM ABTS radical solution and 3 mM potassium persulfate stock solutions were prepared. The working solution was prepared by mixing equal amounts of ABTS and potassium persulfate solutions, which were kept in the dark for 12 h. The mixture was further diluted with methanol to obtain an absorbance of approximately 1 unit at 734 nm in a microplate reader (Bio-Tek Instruments, Inc., Winooski, VT, USA). Then 200 µL of ABTS solution was transferred to the well in the microplate, and 50 µL of sample solution containing 1–10 µg/mL was added to each well. Methanol (50%) was added to control ABTS radical activity. The reduction in absorbance was measured by the ABTS scavenging activity of the tested extract and calculated with Equation (1):

ABTS scavenging activity (%) = [1 − A_sample_/A_control_] × 100 (1)

where A_sample_ is the absorbance in the presence of the sample, and A_control_ is the absorbance without the test sample. The IC_50_ (the concentration of the inhibitor at which ABTS radical was 50% scavenged) was determined graphically.

#### 3.5.2. DPPH Radical Scavenging Activity Assay:

To perform DPPH radical scavenging activity, a stock solution of DPPH radical in methanol was prepared by adding 12 mg in 50 mL methanol which was then stored at −20 °C until the experiments were performed. A working solution was prepared by transferring an aliquot of DPPH radical solution to obtain an absorbance of 1 at 515 nm using the microplate reader. Then, 200 µL of DPPH solution was transferred to the well in the microplate and 50 µL of sample solution containing 5–15 µg/mL was added to the assay. The reduction in the absorbance at 515 nm was measured as the DPPH scavenging activity of the tested extract and was calculated with Equation (2):

DPPH scavenging activity (%) = [1 − A_sample_/A_control_] × 100 (2)

where A_sample_ is the absorbance in the presence of the sample and A_control_ is the absorbance without the test sample. The IC_50_ (the concentration of the inhibitor at which the DPPH radical was 50% scavenged) was determined graphically.

#### 3.5.3. Ferric Reducing Antioxidant Power (FRAP)

For the FRAP assay, stock solutions were prepared using 300 mM acetate buffer, pH 4, 10 mM TPTZ (2,4,6-tripyridyl-s-triazene) in 40 mM HCl and 20 mM FeCl_3_.6H_2_O solutions. A fresh working solution was prepared by mixing 25 mL acetate buffer, 2.5 mL TPTZ solution, and 2.5 mL FeCl_3_.6H_2_O, which was then warmed to 37 °C before use. The working solution (200 µL) was transferred to each well, and 50 µL of sample solution containing 5–15 µg/mL was added. Water was used as the control. A standard Trolox solution was used for the standard curve, and ferric reduction activity was measured and expressed as Trolox equivalent.

#### 3.5.4. ORAC Antioxidant Assay:

The Oxygen Radical Absorbance Capacity (ORAC) assay was performed with 96 well plates by following the method of Ou et al. [46]. Peroxyl radicals were freshly prepared using 2,2′-azobis(2-amidinopropane) dihydrochloride. Fluorescein was used as a substrate. Samples were prepared by adding 0.02–0.03 g in 20 mL 70% ethanol and diluted accordingly. Aliquots of 200 µL of blank, standards, and samples were transferred to the wells in the microplate and 150 µL fluorescein solution was added, followed by 25 µL K_2_HPO_4_/KH_2_PO_4_ buffer (0.75 µM) and then 25 µL AAPH solution. Fluorescence conditions were as follows: excitation at 485 nm and emission at 520 nm. A standard curve was prepared using Trolox as a standard. The fluorescence loss due to free radical damage was measured as fluorescence time/intensity in units of “area under curve” (AUC). Antioxidant capacity was measured as µmol TE/g.

The HORAC assay was performed as described by Mullen et al. [15], using fluorescein as the probe. The area under the fluorescence decay curve was monitored in the presence and absence of WCC, WCCE1, and WCCE2 to calculate the hydroxyl scavenging activity.

The NORAC assay was performed by monitoring the oxidation of DHR-123 with microplate fluorescence reader FL600 (Bio-Tek Instruments, Inc., Winooski, VT, USA), with excitation and emission wavelengths of 485 and 530 nm, respectively, at room temperature as described by Mullen et al. [15].

The SORAC assay was performed following the method of Mullen et al. [15], using hydroethidine as the probe to measure the superoxide scavenging activity. Superoxide radicals were generated by the mixture of xanthine and xanthine oxidase, which showed an absorbance at 586 nm.

The singlet oxygen scavenging assay (SOAC) was performed according to the method of Mullen et al. [15], generating singlet oxygen from the mixture of H_2_O_2_ and MoO_4_^2−^ and using the probe hydroethidine.

### 3.6. Measurement of Enzyme Inhibition Activity

#### 3.6.1. Inhibition of α-Amylase Assay

The α-amylase activity was measured using human salivary amylase as the enzyme source, and a p-nitrophenol substrate analog supplied in the BioVision α-amylase inhibitor screening kit. The enzyme and substrate were reconstituted in 100 µL assay buffer and stored in 10 µL aliquots. Then, 490 µL of assay buffer was added to prepare the working solution. Fifty microliters of sample or water were transferred to the wells of the microplate, followed by the addition of 50 µL of enzyme solution. Samples were covered in aluminum foil and kept for 10 min. Then, 50 µL of substrate solution was added to each well containing the mixture of sample and enzyme. The progress of the reaction was monitored by measuring the absorbance at 405 nm, following the production of p-nitrophenol from the enzymatic reaction. The rate of the reaction in the presence and absence of the inhibitor was determined using the corresponding slopes. The percent inhibition was calculated using Equation (3):

% of relative inhibition = [1 − slope of the reaction in presence of inhibitor/slope of the reaction of control] × 100(3)

The IC_50_ was determined graphically as the concentration of the inhibitor at which 50% of the α-amylase activity was inhibited.

#### 3.6.2. Inhibition of α-Glucosidase Assay

Glucosidase inhibitory activity was measured using α-glucosidase from *Saccharomyces cereviciae* and *p*-nitrophenyl-α-d-glucoside (pNPG) as substrates. A stock solution of glucosidase enzyme was prepared (1 mg) in 1 mL phosphate buffer and diluted 100 times for use as a working solution. The pPNPG was prepared by adding 6 mg of pNPG in 10 mL phosphate buffer. The initial reaction rate of the hydrolysis of pNPG in the absence of an inhibitor was monitored by the continuous production of p-nitrophenol at 405 nm. For this, 50 µL sample solution or buffer (for control) was added to each well followed by 20 µL enzyme solution. The reaction hydrolysis reaction was initiated by adding 10 µL of substrate and 20 µL of buffer solution. The slope of the reaction kinetics was measured in the absence of an inhibitor, which was considered as the control, and in the presence of inhibitors at different concentrations.

#### 3.6.3. Inhibition of Acetylcholinesterase (AChE) Assay

AChE activity was measured following the Ellman method, in which AChE produces thiocholine in reaction with 5,5′-dithiobis (2-nitrobenzoic acid) (DTNB) in the Sigma Aldrich kit [47]. Enzyme solutions were prepared with approximately 1 mg/mL concentration. Working solutions were prepared by transferring 2 µL and making it to a total of 100 µL using assay buffer and then diluting to half. The reaction mixture was prepared by adding 1540 µL buffer, 10 µL substrate, and 5 µL DTNB. To start the reaction kinetics, 45 µL of enzyme solution was transferred to each well and then 5 µL of sample solution or water was added, and 150 µL of reaction mixture was added. The progress of the reactions was monitored by starting the reaction kinetics at 412 nm.

### 3.7. Data Analysis

The results are expressed as mean ± standard deviation (SD) from three replicate experiments using Microsoft Excel 365 ver.1908. A one-way analysis of variance (ANOVA) was used to analyze the data by using Sigmaplot 14.5 (San Jose, CA, USA), and significance was accepted at *p* < 0.05.

## 4. Conclusions

In conclusion, two semi-purified coffee cherry extracts, WCCE1 and WCCE2, were rich in chlorogenic acid and caffeine, with minimums of 40% and 70%, respectively. Investigation of the inhibitory properties on the digestive enzymes viz. α-amylase and α-glucosidase indicated that WCCE1 inhibited their activity in a dose-dependent manner. Moreover, caffeine-enriched coffee cherry extract, WCCE2, showed the strong anti-cholinesterase activity required to treat Alzheimer’s disease. WCCE1 also exhibited strong radical scavenging activities in all antioxidant assays, including DPPH, ABTS, FRAP, and ORAC. Therefore, consumption of chlorogenic acid-enriched coffee cherry extract may have a potential role in regulating postprandial glycemic control. Since caffeine intake alone has some limitations, blending chlorogenic acid and caffeine-rich extracts with a specific ratio may serve as a functional food and minimize the risks involved with diabetes and Alzheimer’s disease. Meanwhile, further in vivo and clinical studies are required to unravel the antidiabetic and anti-Alzheimer’s activities of these extracts.

## Figures and Tables

**Table 1:** Identification and quantification of major chemical compounds in WCC, WCCE1, and WCCE2.

[M−H]-*m*/*z*	[M+H]-*m*/*z*	Compound	MS/MS	Concentration (% *w*/*w*)		
				WCC	WCCE1	WCCE2
353.09	-	3-CQA	135, 179, 191	1.02 ± 0.26	8.19 ± 0.10	1.22 ± 0.00
353.09	-	5-CQA	191	2.95 ± 0.32	14.38 ± 0.07	2.30 ± 0.01
353.09	-	4-CQA	135, 173, 179, 191	1.17 ± 0.16	9.41 ± 0.71	1.31 ± 0.00
367.10	-	5-FQA	173, 191	0.41 ± 0.12	3.38 ± 0.01	0.28 ± 0.00
367.10	-	4-FQA	173, 193, 191	0.18 ± 0.07	2.23 ± 0.02	0.16 ± 0.00
515.12	-	3,4-diCQA	173, 179, 191, 353	0.39 ± 0.17	3.43 ± 0.01	0.33 ± 0.00
515.12	-	3,5-diCQA	135, 179, 191, 353	0.23 ± 0.08	1.8 ± 0.00	0.17 ± 0.00
515.12	-	4,5-diCQA	173, 179, 191, 353	0.41 ± 0.16	3.64 ± 0.01	0.33 ± 0.00
		Total chlorogenic acid		6.76 ± 1.34	46.46 ± 0.93	6.1 ± 0.01
-	138.05	Trigonelline	94	0.4 ± 0.00	2.40 + 0.09	<0.01
-	195.09	Caffeine	138	1.12 ± 0.17	0.90 ± 0.00	73.60 ± 0.65

**Table 2:** DPPH and ABTS radical scavenging activities of WCC, WCCE1, and WCCE2.

Sample	DPPH (IC_50_) (µg/mL)	ABTS (IC_50_) (µg/mL)
WCC	300.00	140.00
WCCE1	11.12	7.85
WCCE2	ND	ND

ND—Not Detectable.

**Table 3:** ORAC 5.0 assay antioxidant activity of WCC, WCCE1, and WCCE2.

	Antioxidant Activity (µmol TE/g)					
Sample	ORAC	HORAC	NORAC	SORAC	SOAC	Total ORAC
WCC	772	764 ± 45.70	128.33 ± 11.15	126.33 ± 10.60	237.66 ± 1 6.56	2028.32
WCCE1	9029	11,089.67 ± 942.59	1547 ± 72.80	76.33 ± 9.45	8623 ± 314.72	30365
WCCE2	ND	230.33 ± 30.03	350 ± 12.12	ND	ND	580.33

ND—Not Detectable.

**Table 4:** The enzyme inhibitory activities of WCC, WCCE1, and WCCE2.

Sample	IC_50_ α-Amylase (mg/mL)	IC_50_ α-Glucosidase (mg/mL)	IC_50_ AChE (mg/mL)
WCC	15.00	33.00	ND
WCCE1	1.74	2.42	ND
WCCE2	ND	ND	0.09
Caffeine	ND	ND	0.065
Chlorogenic acid	2.8	2.1	ND
Acarbose	0.008	1.25	ND

ND—Not Detectable.

## Data Availability

The data presented in this study are openly available from the authors.

## Sample Availability

All samples are available from authors.
